# Tadpole transport logistics in a Neotropical poison frog: indications for strategic planning and adaptive plasticity in anuran parental care

**DOI:** 10.1186/1742-9994-10-67

**Published:** 2013-11-09

**Authors:** Eva Ringler, Andrius Pašukonis, Walter Hödl, Max Ringler

**Affiliations:** 1 Department of Integrative Zoology, University of Vienna, Althanstrasse 14, A-1090 Vienna, Austria; 2 Department of Cognitive Biology, University of Vienna, Althanstrasse 14, A-1090 Vienna, Austria; 3 Department of Tropical Ecology and Animal Biodiversity, University of Vienna, Rennweg 14, A-1030 Vienna, Austria

**Keywords:** Dendrobatidae, *Allobates femoralis*, Natural population, Parental care, Spatial behaviour, Larval transport

## Abstract

**Introduction:**

Individuals should aim to adjust their parental behaviours in order to maximize the success of their offspring but minimize associated costs. Plasticity in parental care is well documented from various bird, mammal and fish species, whereas amphibians were traditionally assumed as being highly instinct-bound. Therefore, little is known about ‘higher’ cognitive abilities of amphibians, such as strategic planning and behavioural flexibility. Dendrobatid frogs have evolved a remarkable diversity of parental behaviours. The most noticeable of these behaviours is tadpole transport, which is obligatory in almost all species. Nonetheless, there is limited knowledge about spatial and temporal patterns of tadpole transport and the possible existence of behavioural plasticity on the individual level. In this study, we investigated correlates of tadpole transport behaviour in a natural population of the dendrobatid frog *Allobates femoralis* during five years.

**Results:**

Tadpole transport was predominantly observed during morning hours. Although tadpoles were carried almost exclusively by males (N = 119), we also observed ten females performing this task. The parentage analysis revealed that in all cases females transported their own offspring. In contrast, four tadpole-carrying males were not the genetic fathers of the larvae they were transporting. The average clutch size of 20 eggs and our observation of an average of 8 tadpoles on the back of transporting individuals indicate that frogs do not carry entire clutches at once, and/or that they distribute their larvae across several water bodies. Contrary to the predictions from a hypothetical random search for deposition sites, the number of transported tadpoles was higher in males that travelled over longer distances.

**Conclusions:**

Our results suggest a strong selective pressure on males to shift the time invested in tadpole transport to periods of low intra-specific competition. The number of tadpoles on the back of the males significantly correlated with displacement distance from the respective home territories, indicating a strategic non-random tadpole transport rather than random search for suitable tadpole deposition sites during tadpole transport. The observation of females who occasionally transported larvae supports the prevalence of adaptive plasticity in parental behaviours even in a species with a rather low level of parental care.

## Introduction

Costs and benefits of parental care play a major role in the evolution of parental behaviours and have been investigated in detail across various animal taxa (for reviews see [1,2]). Parental care is assumed to evolve only in situations where the benefits of care exceed the associated costs [3]. While benefits are mainly derived from an increased survival of offspring, the costs often remain obscure and are difficult to measure. Costs of parental care to the caregiving individual include direct costs, such as an increased predation risk and higher energetic expenditure, as well as indirect costs, such as missed mating opportunities [1,4]; for amphibians see [5]. In many animal species, the certainty of parentage for the caregiving individual/sex has a significant impact on the parental effort provided (likelihood of being the ‘true’ parent; [4,6-10] but see [11]). According to the associated costs and benefits of care, individuals should aim to adjust their parental behaviours in order to maximize the success of their offspring while minimizing the associated costs [12]. These adjustments can either be long-term behavioural changes associated with ‘learning’ or changes in the nervous system as a result of experience (i.e. developmental behavioural plasticity). They can also involve immediate responses to changing conditions (i.e. activational behavioural plasticity; [13]). Activational behavioural plasticity therefore encompasses short-term effects, where the behavioural response is not affected by past environmental conditions but solely by the current situation. In this manuscript we mainly focus on activational behavioural plasticity in our study species.

Amphibians were traditionally assumed to be highly instinct-bound, if not simple ‘reflex machines‘ (cf. [14]). Over the last decades, and as a result of many behavioural and neurophysiological studies, this view has slightly changed [15]. Still, little is known about ‘higher’ cognitive abilities of amphibians, such as strategic planning and behavioural flexibility. Amphibians are particularly interesting for studying the evolution of parental care because all forms (male, female, or bi-parental care) can be found across this taxon. In amphibians, parental care is relatively most common in salamanders and newts, although parental behaviour in these two groups is mainly restricted to the attendance of eggs and larvae [16-18]. Anuran amphibians generally do not provide any care after oviposition. Nonetheless, many frog families have independently evolved at least some form of parental care [16,17,19]. Interestingly, 92% of all anuran species that exhibit parental care deposit their eggs outside of water [17]. The transition towards terrestrial eggs probably promoted the evolution of various strategies to enhance and ensure larval development, given that anuran larvae in general, even those of species with terrestrial eggs, are aquatic and require water to complete metamorphosis [5]. The main adaptations in this context are either direct development of tadpoles inside the clutch [20,21], or parental care for eggs and/or tadpoles [16,17,20]. Parental behaviour can protect the terrestrial eggs from desiccation, pathogens, and predators, and can ensure the development of aquatic larvae until metamorphosis. It may include egg guarding, attendance and provisioning of larvae, transport of eggs, and transport of larvae (reviewed in [5]). In cases where tadpoles must be transported to water, parental care is even obligate for offspring survival. In situations where tadpole deposition sites are not commonly available inside parental territories, the transport of aquatic larvae over larger distances might impose considerable time and energy investments on the transporting parent (but see [22]). Particularly the combination of territoriality and terrestrial egg deposition might have played an important role in the evolution of parental care in frogs. In aquatically breeding species, multiple paternities in single clutches are quite common. This is due to multiple males amplexing single females and due to competing stray sperm from males in neighbouring matings [23]. In terrestrially breeding species, oviposition commonly takes place inside the territories of individuals. This should result in a high certainty of parentage for both males and females [24]. Such a high certainty, in turn, might have led to generally higher parental investment in territorial species.

Poison frogs (Dendrobatidae) show a remarkable diversity in their parental behaviour [25-33]. Male tadpole transport without any previous and/or further provisioning and attendance is assumed to be the ancestral form of parental care in dendrobatid frogs [27,29], but exclusive female- and bi-parental care have evolved in several species (see [34,35]). Most dendrobatids carry their larvae from terrestrial egg deposition sites to water bodies such as small streams, swamps, temporary ponds, or to phytotelmata in leaf axils, bromeliads, or tree holes [5,31]. In some species, larvae even complete their entire development while being carried on the parent’s back [36,37]. For several dendrobatid species, recent research has demonstrated the presence of behavioural plasticity in deposition strategies according to predator presence (visual cues: [38], chemical cues [39]), phytotelm quality [40], seasonal variation in desiccation risk [41], and presence of conspecific tadpoles [42]). We hypothesize that further specific behavioural adaptations have evolved in this taxon to minimize the associated costs of tadpole transport, such as energy investment, predation risk, or lost mating opportunities. All other things being equal, larger individuals should be able to take up more tadpoles at once than smaller ones. They either provide more space for tadpoles to hold on or are physically stronger. If the main costs of tadpole transport arise from indirect negative effects associated with transporting distance, especially time and energy expenditure, then we would expect transporting individuals to take up as many tadpoles as possible, preferably all at once. They may then either deposit all the tadpoles in the first water body they encounter, or distribute them successively over several water bodies along their route as they search for suitable water bodies. These behaviours would either yield no identifiable effect or a negative correlation between the number of tadpoles on the back of transporting individuals and the distance covered. Given that tadpoles are not immediately released when the parent jumps into water, males might be able to influence the actual number of tadpoles released by the duration they spend in the water pool and also by the number of wiping movements of the hind limbs (E. Ringler pers. obs.).

In the present study we used the anuran model species *Allobates femoralis*, a small diurnal poison frog (Dendrobatidae), which is distributed throughout Amazonia [43], to investigate correlates of tadpole transport behaviour. During the reproductive season, which coincides with the local rainy season [44,45], males call from elevated structures on the forest floor to announce territory possession to male competitors and to attract females [46]. Pair formation, courtship, and mating take place in the male’s territory [47,48]. Here, externally fertilized clutches of approximately 20 eggs are laid in the leaf litter [49,50]. Both sexes are highly iteroparous and polygamous within prolonged reproductive periods [51]. Females can produce one clutch every eight days on average [49], and males were observed to attend up to five clutches at the same time [51]. Tadpole transport takes place after 15–20 days of larval development and is mainly performed after heavy rains by males [28]. Nonetheless, occasional cases of transporting females have been documented [49,52-54]. Tadpoles are usually deposited in rather large water bodies ranging from medium-sized temporal pools to floodplains [55,56], as well as peccary wallows and footprints [57]. Small terrestrial phytotelmata such as palm fronds and holes in fallen trees are also used when available (pers. obs. by all authors). Studies from captivity have shown that *A. femoralis* distribute their tadpoles across several water pools, if available, with tadpoles requiring 40–50 days until metamorphosis [49].

In this study, we investigated correlates of tadpole transport behaviour in a natural population of the dendrobatid frog *A. femoralis* during five consecutive breeding seasons. This involved analysing physical properties and spatial behaviour of all individuals in the study population and using molecular parentage analyses to verify parent-offspring relationships.

## Results

During the whole study period from 2008 to 2012, we recorded 1373 individual adult *A. femoralis* (*N*_m_/*N*_f_; 2008: 144/60, 2009: 160/71, 2010: 203/97, 2011: 247/107, 2012: 192/92) in the study area. The survival rates of adult frogs were relatively low: year-to-year recapture rates averaged 14% for males and 15% for females, corroborating previous findings in this and another *A. femoralis* population (cf. [51,56,58]).

We observed a total of 129 tadpole transport events (2008: *N* = 14, 2009: *N* = 18, 2010: *N* = 32, 2011: *N* = 31, 2012: *N* = 34). In the vast majority of cases, males performed this task (92.2%, 119 out of 129). However, we also observed 10 females with larvae on their back (7.8%). Twelve individuals, all of them males, were observed twice during tadpole transport: 4 males in two successive years, and 8 males within the same year. The number of tadpoles carried by individual frogs ranged from 1 to 25 (median ± iqr = 8 ± 2, *N* = 129). The number of tadpoles transported by single frogs did not differ significantly between the sexes (males: median ± iqr = 8 ± 2, range = 1–25, *N* = 106; females: median ± iqr = 9 ± 2, range = 1–17, *N* = 10; Mann–Whitney test, *U* = 523.5, *P* = 0.949).

Tadpole transport occurred mainly during morning hours (median ± iqr = 11:13 ± 10:30, range 8:43–18:35, Figure 1). The time of observations did not differ significantly between males and females (Mann–Whitney test, *U* = 594, *P* = 0.424).

**Figure 1:**
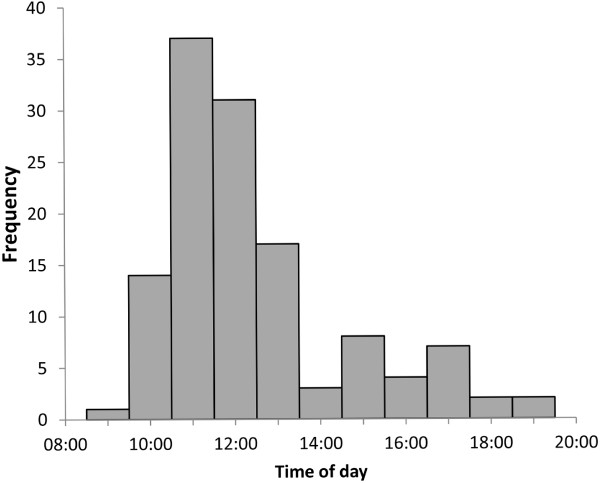
Temporal distribution of observed tadpole transport events.

In the parentage analysis, COLONY always assigned both tested tadpoles from a single transporting event to an identical parent pair. For 115 tadpole-pairs (96.6% out of 119), paternity was assigned to the male carrier, while for four pairs paternity was assigned to either a direct neighbour (*N* = 3; for a definition of direct neighbour see [50]) or an unidentified male (*N* = 1). For the ten tadpole pairs transported by females, maternity was in all cases assigned to the female carrier. During tadpole transport, males were encountered on average 27.52 ± 30.90 m (median ± iqr) away from their home territories (range = 1.64–185.14 m).

The multiple correlation analysis identified a significant correlation between ‘age’ and snout urostyle length ‘SUL’ (Spearman, *ρ* = 0.306, *P* = 0.003, *N* = 95; Table 1). Accordingly, ‘age’ was omitted in the subsequent analysis, given that SUL offers the higher explanatory power.

**Table 1:** Multiple Correlation Analysis of the variables ‘age’, ‘time of day’, ‘SUL’, and ‘displacement distance’ (‘dist_log’)

		**Dist_log**	**Age**	**Time**	**SUL**
Dist_log	ρ		−0.196	0.158	0.192
	Sign. (two tailed)		0.081	0.140	0.082
	*N*		80	89	83
Age	ρ	−0.196		−0.137	**0.306**
	Sign. (two tailed)	0.081		0.173	**0.003**
	*N*	80		100	**95**
Time	ρ	0.158	−0.137		−0.084
	Sign. (two tailed)	0.140	0.173		0.395
	*N*	89	100		104
SUL	ρ	0.192	**0.306**	−0.084	
	Sign. (two tailed)	0.082	**0.003**	0.395	
	*N*	83	**95**	104	

ρ (Spearman correlation coefficient), *P* (significance, two tailed), *N* (number of tested cases), significant correlations are given in bold.

**Figure 2:**
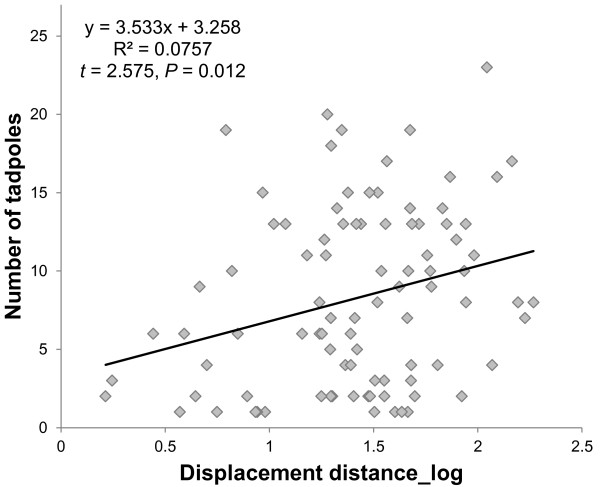
**Relation between ‘number of tadpoles’ and ‘displacement distance’ of male *A. femoralis*.**

The stepwise linear regression model revealed that only displacement distance significantly predicted the number of tadpoles on a male’s back (*t* = 2.575, *P* = 0.012) (Table 2, Figure 2), but not time of day (*t* = −0.622, *P* = 0.536) or SUL (*t* = 1.101, *P* = 0.274).

**Table 2:** Output table of the stepwise multiple linear regression analysis

***Predictor***	***β***	***t***	***p***
Distance	3.810	2.575	0.012
Time of day	−0.067	−0.622	0.536
SUL	0.119	1.101	0.274

We found a significant correlation only for the parameters ‘number of tadpoles’ and ‘displacement distance’, but not for ‘time of day’ and ‘SUL’. Model statistics: R^2^ = 0.076, F(1,81) = 6.63, *p* = 0.012.

## Discussion

The present study underlines the existence of adaptive plasticity in parental behaviour in amphibians by comprehensively analysing the tadpole transport behaviour in *A. femoralis* during five years. Our data contribute to knowledge on tadpole transport behaviour in other dendrobatid species and improve our understanding of the evolution of male/female parental care in poison frogs.

The average displacement distance of *A. femoralis* males from their home territories was 27 m, which is about double the diameter of their average territories (13.9 m, cf. [59]). One transporting male travelled almost 185 m (straight line distance) and even crossed a small creek. This indicates that suitable water bodies for larval deposition are not easily found within the territories of all males, potentially constituting a limiting resource in our study population. Tadpole transport was mainly observed during morning hours (Figure 1). We attribute this to several factors. On the one hand, the maximum calling activity in *A. femoralis* takes place in the afternoon in our study population (3 to 6 p.m., pers. obs. by all authors) and elsewhere [45]. Thus, males that carry their tadpoles in the morning will typically have returned to their home territories by afternoon (cf. [60]), minimizing the risk of losing mating opportunities. Particularly if distances to suitable aquatic sites are far, we expect a strong selective advantage for males that shift tadpole transport to times with low or no conspecific calling activity. On the other hand, temperature is slightly lower during morning hours, potentially causing less energy expenditure than afternoon transporting (cf. [61]). Finally, tadpole transport in the morning provides males with more daylight hours to return to their home territory.

Previous studies have already mentioned that *A. femoralis* females occasionally transport tadpoles [49,52-54]. However, concise data on the frequency of this behaviour under natural conditions were lacking. In our study almost 8% of all transporting events were performed by females. The parentage analysis revealed that all these females carried their own offspring. Females typically choose their mates within 20 m of their perching sites and return to their resting sites immediately after oviposition [47,50,56]. It is therefore very unlikely that females ‘accidentally’ end up with tadpoles on their back unless they actively return to the oviposition site, sit on the clutch, and wait until the tadpoles climb on her back, which takes about 30 min (E. Ringler pers. obs. in males). Nonetheless, during the five-year study we never observed females returning to their previously laid clutches when males were still present. Consequently, we see no indication that female transporting behaviour could be an infrequently expressed error reflecting similar male/female nervous and endocrine systems (cf. [62]). The question remains how such behaviour could become adaptive; i.e. how benefits exceed costs. We hypothesize that the costs of tadpole transport might be less for females than for males. Females do not defend territories and thus do not risk losing a territory during their absence. Furthermore, under optimal conditions, they can produce a clutch every eight days [49]. Oviposition is not restricted to a limited time frame and is often triggered by specific reproductive stimuli (cf. [63]). Accordingly, we assume that tadpole transport would not severely restrict mating opportunities. Energy expenditure and predation risk are presumably the same for males and females. At the same time, females could ensure further survival of a clutch in which they have already invested substantial time and energy (and that already survived over two weeks). We therefore also hypothesise that, due to the low tadpole transport costs, females might gain substantial fitness benefits by flexibly taking over parental duties if the male is absent. In other taxa, behavioural flexibility with respect to parental care has generally been studied in bi-parental species. When both parents are involved in brood care, males and females often cooperate by exhibiting different parental roles [64]. Such sex-specific parental behaviours, however, might change when one of the parents disappears. Manipulation experiments in fish and birds have shown that widowed parents were capable of raising the offspring alone by either increasing the own parental effort or even switching between parental roles [65,66]. These experiments suggest that the coordination of roles displayed in bi-parental species is flexible and may depend on the presence and the behaviour of the other parent. Little attention has been paid to such flexibility in uni-parental species, i.e. the takeover of parental duties in the generally non-caring sex. Behavioural plasticity of tadpole deposition behaviour has been documented for some dendrobatid species in several contexts [38-42]. These frogs integrate multiple factors such as water quality, presence of predators, con- or heterospecific tadpoles, and pool size and adapt their behaviour accordingly [33]. Further studies are needed to reveal reasons for and mechanisms of female tadpole transport in *A. femoralis*.

Surprisingly, not all transporting males were identified as the genetic fathers of the carried larvae. Three males carried the larvae of immediate neighbours (i.e. calling males of adjacent territories), one the larvae of an unidentified individual (i.e. the genotype of the putative father as inferred by COLONY was not found amongst our paternal candidates). In the former three males, we assume that territory shifts and/or territory overlaps might have occurred. Such shifts and overlaps are rare during the breeding season (cf. [56,59]), but they might occur due to changes in forest floor structure (e.g. fallen branches or trees) or interactions amongst competing males. In the one case where no suitable father could be identified, we speculate that the actual father died or abandoned his territory prior to our study period and that another male had taken over his territory. Another possibility would be that the clutch was sired by a sneaking male (cf. [67]), although we never directly observed any active sneaking behaviour during our five-year study ([51], pers. observations of all authors), or found indirect evidence through genetic parentage inferences of larvae from clutches [50]. We assume that successful siring of clutches by sneaking males is largely precluded by the elaborate courtship behaviour and because mating takes place in vigorously defended territories [56,68].

Perhaps there is a fixed behavioural pattern in *A. femoralis* males to carry all encountered clutches inside their territory to water sites without distinguishing between own or foreign offspring. Although such behaviour would facilitate the evolution of sneaking behaviour, we have no indications that sneaking is a frequent alternative mating strategy in this species. Further experimental studies are needed to reveal if a fixed action pattern is involved and whether it is exclusively elicited in males that simultaneously have own clutches inside their territories.

Adult frogs carried an average 8 larvae on their back. In a Brazilian *A. femoralis* population, frogs carried all tadpoles from single clutches at once [48]. In *A. femoralis*, clutches average about 20 eggs and larval mortality inside the clutch is low [49,50]. The low average number of carried tadpoles in our study indicates that these frogs distribute their larvae across several water bodies and probably do not transport entire clutches at once to single pools.

The distances covered by *A. femoralis* males were considerably longer than those reported for any other dendrobatid frog (*Dendrobates pumilio*: max. 20 m, I. Meuche and H. Pröhl pers. comm.; *Ranitomeya ventrimaculata*: within a circle with a maximum diameter of 8.5 m, approximated from the area of the maximum 95% home range kernel, [69]). Given our sampling regime of registering all frogs at chance encounter locations along their transportation route, the maximum transporting distances must be considered low estimates. Surprisingly, the distance of males to their home territories during tadpole transport correlated significantly positively with the number of tadpoles on their back. No correlation was found between tadpole number and SUL or time of day. The high variance of data points in the correlation between ‘number of tadpoles’ and ‘displacement distance’ (Figure 2) is probably because individual frogs were caught at chance locations along their transport route. We therefore hypothesize that the number of tadpoles taken up at once is influenced by the distance of the territory to suitable water bodies (cf. [49]). This implies a detailed spatial knowledge of the surrounding area along with some sort of strategic planning rather than random searching. Given that deposition sites are not abundant and unevenly distributed in our study area, knowledge about pool locations would significantly reduce effort and risks of tadpole transport. While in *Dendrobates auratus* males regularly explore their surroundings for potential deposition sites [70], *A. femoralis* males generally do not leave their home territories during the reproductive season [56]. However, they might have collected spatial information during the onset of the rainy season before territories become established, or during previous tadpole transport events. A recent study revealed a high homing performance of male *A. femoralis* after experimental translocations [71]. Translocated males successfully returned to their territories from up to 400 m and showed a very high homing success for distances up to 200 m, suggesting that this ability is restricted to an area of potential familiarity.

Several direct and indirect costs of tadpole transport have been proposed (reviewed in [5]). Predation risk might increase if larval transportation reduces movement ability (but see [72]). We lack information on whether this is the case in *A. femoralis*. Furthermore, if foraging is reduced or absent while transporting larvae, frogs might significantly lose weight, posing severe health risks or disadvantages in subsequent male-male competitions. Durations of tadpole transport in dendrobatid species are highly variable (see table 11.3, p. 524–526 in [5]). Some species release the tadpoles within a few hours (*D. pumilio*, [30]), while in others the larvae remain on the back for several days (e.g. *Colostethus inguinalis*, [26]). *A. femoralis* is an opportunistic feeder, suitable food is quite abundant and tadpole transport generally takes only a few hours [60] – male *A. femoralis* were even observed to feed during acoustic playback experiments under experimental conditions [73]. We therefore do not assume any severe weight loss during tadpole transport in this species. Further costs include the invested time and energy. We observed individual frogs to carry up to 25 tadpoles, corresponding to about 28% of their own body weight (mean_tadpole_ = 0.0192 g, *N* = 3; mean_males_ = 1.7 g, *N* = 121, unpubl. data). The energy expenditure in tadpole-transporting frogs is no doubt higher than unencumbered movement, but has not been investigated in any dendrobatid species so far. The factor ‘time investment’ could also impact individual fitness. For example, mating opportunities might be lost due to the absence from home territories or resting sites. Particularly in cases of strong male-male competition for territories, there may be strong selective pressure against male tadpole transport, given the high risk of losing the territory while absent (cf. [74]). Nevertheless, in dendrobatid frogs, male-only care is quite widespread and is assumed to be the ancestral state of parental care in the whole family [27,29]. We suppose that these indirect costs in *A. femoralis* males are minimized because tadpole transport mainly occurs during morning hours, when less intra-specific competition for territories or courtship takes place. Nonetheless, possible negative effects of high mating success and subsequent increased parental effort in *A. femoralis* males (cf. [75,76]) remain to be investigated.

## Conclusions

The present study investigates the tadpole transport behaviour in a Neotropical frog with male territoriality and paternal care. Tadpoles were transported mainly during morning hours, indicating a selective pressure on males to shift the invested time towards periods of low intra-specific competition. The number of carried tadpoles significantly correlated with displacement distance from the respective home territories. This suggests strategic non-random transport rather than random search for suitable deposition sites in *A. femoralis* males. Females occasionally transported their own offspring, supporting an adaptive plasticity in parental behaviours, even in a species with a low level of parental care. These observations combined indicate strategic planning and behavioural flexibility in our study species, two poorly known behavioural phenomena in anuran amphibians.

## Methods

### Study population

We conducted our study in a lowland rainforest near the field camp ‘Saut Pararé’ (4°02′ N, 52°41′ W) in the nature reserve ‘Les Nouragues’, French Guiana, in a natural population of *A. femoralis*. The study plot was approximately 180 m × 450 m in size and naturally delimited by a river and two streams (for more details see [51]). Sampling took place during the reproductive period of *A. femoralis* in the years 2008 to 2012 (28 January until 24 April 2008, 17 January until 16 March 2009, 16 January until 16 March 2010, 30 January until 24 February 2011, and 27 January until 5 April 2012). We conducted daily surveys from about 0900 h to 1900 h. By continuously sampling all individuals encountered during the surveys, we attempted total sampling of all male and females in the study plot and to record as many individuals as possible in all years. All frogs were identified based on digital photographs of their distinct ventral patterns and sexed by the presence (males) or absence (female) of vocal sac folds. We recorded the precise spatial locations of all frogs in the field on a digital map with the mobile GIS software ArcPad™ 7/8/10 (ESRI), using pocket computers (Hewlett Packard iPaq™ hx1950 & hx4700, Ashtech MobileMapper™ 6) and further handled the data in ArcGIS™ 9.3 (ESRI). We determined body size of all adults (SUL, snout urostyle length) from dorsal photographs in front of a reference scale using the software Image J 1.47 [77]. If individuals were encountered during tadpole transport, we recorded the number of tadpoles on the back, and two tadpoles were taken and preserved in 96% ethanol. All sampling was conducted in strict accordance with current French and EU law and followed the current ASAB guidelines for the treatment of animals in behavioural research and teaching. Detailed descriptions of the sampling procedures for tissue material of adult individuals are given in [51,58].

### Genotyping and parentage analysis

Genomic DNA was isolated using a Proteinase K digestion followed by a standard phenol-chloroform protocol. PCR amplification of seven polymorphic microsatellite loci, genotyping and checking of genotyping errors followed the procedures described in [51]. Genotypes of adults from 2008 and 2009 were already available from a previous study [51]. To identify the parents of the tadpoles sampled in the present study, we used an identical approach. All parentage assignments were performed with the software COLONY v.2 [78]. Each tadpole was tested ‘naïvely’ without prior information about assumed full sib relationships (for tadpoles taken from the same transporting adult) or about assumed parents (i.e. the transporting individual).

### Analyses of tadpole transport activity

We included information of all tadpole transport events recorded over the study period. Consecutive tadpole transport events of the same male (female) were assumed to be independent when the respective larvae were assigned to different mothers (fathers) or when the two successive observations were separated by more than 2 days. If individuals were observed twice within one transporting event, we only included the data of the first encounter in our analyses.

For each observed transporting event, we recorded the parameters ‘displacement distance’ , ‘SUL’ , ‘age’ and ‘time of day’. The parameter ‘displacement distance’ could be assessed only for male tadpole transport events because only in males do the location of their clutch and their own permanent location (i.e. their territory) correspond over lengthier periods. Accordingly, we determined the centroid point of all encounter locations of a given male, assuming that all transporting males had been territorial; reproductive success in *A. femoralis* is significantly associated with male territoriality [51]. We excluded all encounters during tadpole transport from the determination of male territories. Then we calculated the straight line distance of the centroid points to the respective encounter locations during tadpole transport. Males exclusively observed during transport were excluded from the analysis. When an apparent territory change occurred within one sampling period, we included only the territory that was temporarily closest to the respective transport event to calculate transporting distance. For the subsequent multiple stepwise regression analysis, we log-transformed the transport distances because the number of tadpoles transported is limited by clutch size and cannot increase indefinitely with transporting distance. For the parameter ‘age’ , we differentiated between ‘new’ (all frogs that were first encountered in the respective year of sampling) and ‘old’ individuals (frogs already encountered in previous years). We did not include individuals from the first year (2008) because survivors from previous years could not be identified. ‘Time of day’ corresponds to the exact time of the respective tadpole transport event, converted into minute of the day (i.e. 12:00 a.m. = 720 min).

Prior to the stepwise multiple regression analysis to test possible predictors of the number of transported tadpoles, we performed a multiple correlation analysis to identify possible co-linearity of the tested variables. Finally, we performed a stepwise linear regression analysis with ‘displacement distance’ and ‘SUL’ as potential predictors of the number of tadpoles transported. To exclude a possible sampling bias in respect to the time of day when individual frogs were caught, we also included ‘time of day’ in the analysis. In cases where multiple transportation events were observed for single males within one or between successive years, we included only the first transport event per male in order to avoid pseudo-replication.

In the course of a concurrent study on the influence of reproductive resource supplementation on population size, we installed artificial water pools in the study area in March 2009 (Ringler et al., submitted). In order to test whether data points from 2008–2009 and 2010–1012 could be pooled despite altering resource availability, we performed an ANCOVA for all tested correlations to identify potential differences in pre- and post-treatment tadpole transportation behaviour. There was no significant difference in the relation number of tadpoles ‘tp’ and all tested variables before and after the installation of the artificial pools (tp_distance: *F*_23,68_ = 0.005, *P* = 0.946; tp_SUL: *F*_21,84_ = 0.733, *P* = 0.394; tp_time: *F*_27,86_ = 0.698, *P* = 0.405). We therefore pooled all data points in the subsequent analyses.

All statistical analyses were performed in IBM SPSS statistics 20.0.0. Normality of the data was tested with the Kolmogorov-Smirnov *Z* test, and in cases where variables significantly deviated from a normal distribution, non-parametric tests (Mann–Whitney *U* test, Spearmans-Rank-Correlation) were applied.

## Competing interests

The authors declared that they have no competing interests.

## Authors’ contributions

ER and MR designed the study. All authors participated in the field work. ER performed all molecular and statistical analyses and drafted the manuscript. AP, WH and MR provided valuable comments on the manuscript. All authors read and approved the final manuscript.
